# Methicillin-resistant *Staphylococcus aureus* nasal carriage among patients receiving hemodialysis in Taiwan: prevalence rate, molecular characterization and de-colonization

**DOI:** 10.1186/1471-2334-12-284

**Published:** 2012-11-01

**Authors:** Yu-Chuan Kang, Wei-Chen Tai, Chun-Chen Yu, Je-Ho Kang, Yhu-Chering Huang

**Affiliations:** 1 College of Medicine, Chang Gung University, Kweishan, Taoyuan, Taiwan; 2 Departments of Nephrology, Chang Gung Memorial Hospital at Linko, Kweishan, Taoyuan, Taiwan; 3 Department of Nephrology, Yang Ming Hospital, Pingzhen, Taoyuan, Taiwan; 4 Departments of Pediatrics, Chang Gung Memorial Hospital at Linko, Kweishan, Taoyuan, Taiwan

**Keywords:** Methicillin-resistant *Staphylococcus aureus*, Nasal colonization, Hemodialysis, Decolonization, Taiwan

## Abstract

**Background:**

*Staphylococcus aureus*, particularly methicillin resistant (MRSA), is a common pathogen among patients receiving hemodialysis. To evaluate nasal carriage, molecular characterization and effectiveness of decolonization of MRSA among patients receiving hemodialysis in Taiwan, we conducted this study.

**Methods:**

From January to June 2011, two nasal samplings with a 3-month interval were obtained from patients undergoing hemodialysis in a medical center (CGMH), and in a local hospital (YMH) and sent for detection of MRSA. For MRSA carriers, decolonization procedures were administered. All patients in CGMH were observed if MRSA infections occurred during the study period.

**Results:**

A total of 529 nasal specimens (265 from CGMH and 264 from YMH) were collected from 296 patients (161 from CGMH and 135 from YMH). 233 patients participated in both surveys. Average one-time point MRSA carriage rate was 3.8%, and the rate was up to 6.9% for those with two-time point surveys. No additional significant factor for MRSA carriage was identified. Seventy percent of the 20 colonizing MRSA isolates, though categorized as healthcare-associated strains epidemiologically, shared common molecular characteristics of the local community-associated strains. Only one of the 20 MRSA-colonized patients failed decolonization and had persistent colonization, while without any intervention, 17 (61%) of 28 patients with methicillin-sensitive *S. aureus* colonization in the first survey had persistent colonization of a genetically indistinguishable strain. Within the study period, two patients (1.24%) in CGMH, one with MRSA colonization (9.1%), developed MRSA infection.

**Conclusion:**

A substantial proportion of patients receiving hemodialysis in Taiwan had MRSA colonization, mostly genetically community strains. Decolonization procedures may effectively eliminate MRSA colonization and might reduce subsequent MRSA infection in these patients.

## Background

Methicillin-resistant *S. aureus* (MRSA) strains were recently classified as two groups by epidemiologic as well as molecular characteristics [1], namely community-associated (CA)- and healthcare-associated (HA)-MRSA. CA-MRSA isolates are usually less resistant than HA-MRSA isolates. The transmission of CA-MRSA clones to healthcare facilities was not only reported in the U.S.A [2,3], but also in Taiwan [4].

Among patients with end-stage renal disease (ESRD), bacterial infections are the major cause of morbidity and mortality during receiving hemodialysis [5] and *Staphylococcus aureus*, particularly methicillin-resistant, is one of the most common pathogens [6,7]. Patients receiving hemodialysis had a significantly higher risk of invasive Staphylococcal infection than normal population [8,9]. Since colonization of *S. aureus* usually precedes clinical infection [10,11], we wondered if the patients receiving hemodialysis have a higher rate of MRSA carriage. In addition, decolonization of MRSA carriage with topical mupirocin and chlorhexidine bathing has been well discussed [12-14] recently, but inconclusive, including in the patients receiving hemodialysis. Hence, we conducted a study to evaluate the carriage rate of MRSA, molecular characterization of these MRSA isolates as well as the effectiveness of decolonization procedures among the patients receiving hemodialysis in both a medical center and a local hospital in Taiwan.

## Methods

This study was approved by the institutional review board of Chang Gung Memorial Hospital and a written informed consent was obtained from each subject.

### Hospitals and patients population

The study was conducted in two hospital-based outpatient hemodialysis clinics at Chang Gung Memorial Hospital (CGMH) and Yang Ming Hospital (YMH) from January 2011 to June 2011. Both hospitals are situated in northern Taiwan. CGMH is a university-affiliated medical center with 51 beds in the outpatient hemodialysis clinic while YMH is a local hospital, providing primary care, with 36 beds in outpatient hemodialysis clinic. In January 2011, all 290 and 150 patients receiving maintenance hemodialysis at CGMH and YMH, respectively, were eligible for and invited to participate in this study. Nasal samplings from the participants were conducted in January (first survey) and March (second survey), 2011, respectively. Only the study patients in CGMH were observed if MRSA infections occurred during the study period.

### Data collection

To identify the potential risk factors for MRSA acquisition, the following information were collected from medical records of the participants at both hospitals: demographics, underlying disease, latest hospitalization, length of time on hemodialysis, blood access of dialysis (Hickman, arteriovenous fistula, Gortex), previous *S. aureus* infection, and usage of other catheters (Foley and tracheostomy tube).

### Microbiologic methods

Each nasal swab was circled the anterior 1 cm of the nasal vestibule of both participant’s nares. The samples, then, were placed into the transport medium (Venturi Transystem, Copan Innovation Ltd.) immediately. Swab samples were inoculated by streak plate method onto Trypticase soy agar with 5% sheep blood plates. Those plates were incubated at 37 degree Celsius overnight. Identification of *S. aureus* was done by conducting morphology, gram stain, and coagulase tests of strains grown on agar plates. To identify MRSA, cefoxitin disk was used by disk-diffusion method according to the recommendation of Clinical and Laboratory Standard Institutes [15].

### Antimicrobial susceptibility study

The antimicrobial susceptibility of all MRSA isolates to 10 antibiotics, including oxacillin, trimethoprim/sulfamethoxazole (SXT), penicillin, teicoplanin, linezolid, clindamycin, doxycyclin, fusidic acid, vancomycin, and erythromycin, was tested in accordance with the guideline of Clinical and Laboratory Standard Institutes [15] by using the disk-diffusion method.

### Molecular characterization

All the MRSA isolates were molecularly characterized, and the molecular methods used included pulsed-field gel electrophoresis (PFGE) with *Sma*I digestion, staphylococcal chromosomal cassette *mec* (SCC*mec*) typing [16], multilocus sequence type (MLST), and *spa* gene typing [17]. In addition, the presence of Panton-Valentine leukocidin (PVL) genes [18] was also examined. The details of the procedures were described elsewhere previously [16-21]. The genotypes of PFGE were designated, as in our previous studies [19,20], in alphabetical order; any new type, if identified, was designated consecutively. PFGE patterns with < 4-band differences from an existing genotype were defined as subtypes. Two isolates were considered to be indistinguishable, highly related, or distinct if they had the same subtype (no band difference), the same genotype (< 4-band differences), or a different type (≥ 4-band differences), respectively. MLST was examined for selective strains of representative PFGE patterns [21].

For those with MSSA isolates from both surveys, PFGE method was used to examine the genetic relatedness of paired isolates from the same subject.

### Intervention and follow-up

Decolonization procedures, intranasal mupirocin ointment twice daily and 2% chlorhexidine shampoo once daily for five days [13,14], were applied to the patients with MRSA colonization after identified. After the decolonization procedures, a follow-up sampling from the nares was obtained one week later.

### Statistical analysis

Comparing MRSA colonization between two hospitals was done using Pearson’s chi-square tests. Continuous variables were compared between patients with MRSA colonization versus patients without MRSA colonization using Student *t* test. The categorical variables were analyzed by chi-square test or Fisher’s exact test, as appropriate. Odd ratios (ORs) were also calculated with 95% confidence intervals (CIs). The definition of statistical significance was p <0.05. For statistical analysis, SPSS 17.0 software was used.

## Results

A total of 529 nasal specimens (265 from CGMH and 264 from YMH) were collected from 296 patients (161 from CGMH and 135 from YMH). Totally, 104 subjects in CGMH and 129 subjects in YMH participated in both surveys. 116 patients in CGMH participated in the first survey, among whom 12 patients withdrew for the second survey, while additional 45 patients participated in the second survey. 129 patients in YMH participated in both surveys, and additional 6 patients participated in the second survey.

The prevalence rates of MSSA and MRSA carriage among the subjects are shown in Table 1. The overall nasal MSSA and MRSA colonization rate was 11.7% and 4.2%, respectively in CGMH and 12.5% and 3.4%, respectively in YMH. The carriage rates for the patients receiving both surveys were similar by batch survey but the rate of MSSA and MRSA carriage was up to 15.9% and 6.9%, respectively, if either specimen from each subject was positive (Table 1). No significant difference was found in terms of nasal MSSA and MRSA carriage rate between two hospitals.

**Table 1:** **Nasal carriage of methicillin-resistant *S. aureus *(MRSA) and methicillin-susceptible *S. aureus *(MSSA) among patients receiving hemodialysis in Taiwan, stratified by patients receiving individual survey or both surveys**

**Colonization**	**CGMH**		**YMH**		**Total**	
	**Individual**	**Both**	**Individual**	**Both**	**Individual**	**Both**
**Subject No. of 1**^**st**^**sampling**	116	104	129	129	245	233
** MSSA**	14 (12.1)	12 (11.5)	16 (12.4)	16 (12.4)	30 (12.2)	28 (12)
** MRSA**	6 (5.2)	6 (5.8)	3 (2.3)	3 (2.3)	9 (3.7)	9 (3.9)
**Subject No. of 2**^**nd**^**sampling**	149	104	135	129	284	233
** MSSA**	17 (11.4)	10 (9.6)	17 (12.6)	17 (13.2)	34 (12.0)	27 (11.6)
** MRSA**	5 (3.4)	3 (2.9)	6 (4.4)	5 (3.9)	11 (3.9)	8 (3.4)
**Total No. of samplings**	265^a^	104^b^	264^a^	129^b^	529^a^	233^b^
** MSSA**	31 (11.7)	13^**c**^ (12.5)	33 (12.5)	24^**c**^ (18.6)	64 (12.1)	37^**c**^ (15.9)
** MRSA**	11 (4.2)	8^**c**^ (7.7)	9 (3.4)	8^**c**^ (6.2)	20 (3.8)	16^**c**^ (6.9)

CGMH: Chang Gung Memorial Hospital, YMH: Yang Ming Hospital, ^**a**^by samplings number, ^**b**^by subjects number, ^**c**^at least one positive specimen.

No significant difference was found for carriage rate of MSSA or MRSA between two hospitals by Fisher’s exact test.

The comparison of demographics and clinical characteristics between patients with and without MRSA colonization are shown in Table 2. No significant factors associated with MRSA colonization was identified, including sex distribution, age distribution, duration of dialysis, underlying disease, blood access etc., neither was MSSA colonization. The data from CGMH only also showed that no significant difference was observed in terms of previous MSSA or MRSA infection within one year, and antibiotics usage within one month (detailed data not shown).

**Table 2:** **Demographics and clinical characteristics of hemodialysis patients with and without methicillin-resistant *S. aureus *(MRSA) colonization**

**Demographic and clinical data**	**No. (%) of subjects**			**Odds ratio**	**95% Confidence interval**	***p *value**^**a**^
	**Total (n=296)**	**MRSA (n=19)**	**Non-MRSA (n=277)**			
**Male**	136(45.9)	6(31.6)	130(46.9)	0.522	0.193~1.413	0.194
**Age**						
19-29	6(2.0)	0	6(2.1)	0.934	0.906~0.963	1.000
30-59	155(52.4)	10(52.6)	145(52.3)	1.011	0.399~2.566	0.981
≥60	135(45.6)	9(47.4)	126(45.5)	1.079	0.425~2.737	0.873
**Underlying diseases**						
DM	125(42.2)	11(57.9)	114(41.2)	1.966	0.767~5.041	0.153
Hypertension	194(65.5)	16(84.2)	178(64.3)	2.920	0.830~10.268	0.081
HBV carrier	32(10.8)	0	32(11.6)	0.928	0.897~0.960	0.241
HCV carrier	46(15.5)	2(10.5)	44(15.9)	0.620	0.138~2.781	0.406
Liver cirrhosis	8(2.7)	0	8(2.3)	0.934	0.905~0.963	1.000
Gastric ulcer	92(31.1)	5(26.3)	87(31.4)	0.780	0.272~2.234	0.643
History of GI bleeding	26(8.8)	3(15.8)	23(8.3)	2.071	0.562~7.635	0.226
Asthma	2(0.7)	0	2(0.7)	0.935	0.908~0.964	1.000
History of TB infection	5(1.7)	0	5(1.8)	0.935	0.907~0.964	1.000
COPD	16(5.4)	0	16(5.8)	0.932	0.903~0.962	0.610
Cancer	25(8.4)	1(5.3)	24(8.7)	0.586	0.075~4.580	1.000
**Current disease**						
Pneumonia	3(1.0)	0	3(1.1)	0.935	0.907~0.964	1.000
URTI	51(17.2)	4(21.1)	47(17.0)	1.305	0.415~4.108	0.752
**Other risk factors**						
Hospitalization ^**c**^	95(32.1)	4(21.1)	91(35.0)	0.545	0.176~1.689	0.287
Previous *S. aureus* infection ^**c,d**^	42(14.2)	4(21.1)	38(13.7)	1.677	0.529~5.323	0.325
*S. aureus* skin infection ^**c,d,e**^	13(4.4)	1(5.3)	12(4.3)	1.227	0.151~9.970	0.586
Previous catheter related infection^**c**^	34(11.5)	3(15.8)	31(11.2)	1.488	0.410~5.397	0.467
Using of immunosuppressant	18(6.1)	2(10.5)	16(5.8)	1.919	0.407~9.039	0.324
Alcohol drinking	24(8.1)	0	24(8.7)	0.930	0.900~0.961	0.381
**Average duration of HD(year)**^**b**^	7.03±0.35	5.60±1.28	7.13±0.36			0.289
Duration > 3 years	11(57.9)	195(70.4)	206(69.6)	0.578	0.224~1.490	0.252
Duration > 5 years	8(42.1)	152(54.9)	160(54.1)	0.598	0.233~1.533	0.280
**Blood access**						
Hickman	22(7.4)	1(5.3)	21(7.6)	0.677	0.086~5.326	1.000
A-V fistula	180(60.8)	11(57.9)	169(61.0)	0.879	0.342~2.254	0.788
Gortex	94(31.8)	7(36.8)	87(31.4)	1.274	0.485~3.347	0.623
**Other catheter**^**f**^	8(2.7)	2(10.5)	6(2.2)	5.314	0.997~28.331	0.087

DM: diabetes mellitus, HBV: hepatitis B virus , HCV: hepatitis C virus, GI: gastrointestinal, TB: tuberculosis, COPD: chronic obstructive pulmonary disease, URTI: upper respiratory tract infection, inf.: infection, HD: hemodialysis, A-V: arterial venous.

^**a**^ For categorical variables, Fisher’s exact test was used for extreme proportions (expected count <5) instead of Pearson’s chi-square test.

^**b**^ data was presented as the mean value ± standard error of the mean for continuous variables. Performed by Student *t* test.

^**c**^ any event happened in one year before sampling.

^**d**^ including general *S. aureus* infection without known susceptibility or resistance of antibiotics.

^**e**^ including impetigo, furuncle, carbuncle, cellulitis, and abscess.

^**f**^ including Foley and tracheostomy tube.

Twenty MRSA isolates from 19 subjects were available for molecular characterization and are shown in Table 3. Six PFGE patterns were identified. All isolates carried either SCC*mec* type IV, V, or VT. PFGE pattern D/sequence type (ST) 59 or 338/*spa* type 437/SCC*mec* V_T_ and PFGE pattern C/ST 59/*spa* type 437/ C with *spa* gene type 437/SCC*mec* IV were the two most common clones, both belonging to endemic community-associated (CA) clones in Taiwan. All the MRSA strains were resistant to penicillin and susceptible to linezoid, teicoplanin, and vancomycin. The susceptibility rates to erythormycin, doxycyclin, clindamycin, trimethoprim- sulfamethoxazole (TMP-SMX), and fusidic acid were 30%, 95%, 40%, 95%, and 90%, respectively.

**Table 3:** **Distribution of pulsed-field gel electrophoresis (PFGE) patterns and other molecular characterization of all 20 methicillin-resistant *S. aureus *isolates**

**PFGE pattern**	**No. (%) of isolates**	**SCC*mec *type**	**Presence of PVL genes**	**MLST type**	***spa *gene type**
** C**	5 (25)	IV	1	59	t0437
** D**	9 (45)	V_T_	8	59,338	t0437
** AG**	1 (5)	IV	0	30	t019
** BR**	1 (5)	IV	0	8	t008
** BM**	3 (15)	V	0	45	t1081
** U**	1 (5)	IV	0	573	t3525

SCC*mec,* staphylococcal chromosomal cassette *mec*; MLST, multilocus sequence type; PVL, Panton-Valentine leukocidin.

After the first survey, all 9 MRSA carriers received decolonization procedures and follow-up samplings revealed negative for MRSA in eight carriers. After the second survey, all 11 MRSA carriers were successfully decolonized. The carrier without successful decolonization after the first survey still had MRSA colonization in the second survey and successfully decolonized subsequently. For this patient, the two isolates from both surveys were indistinguishable and characterized as ST338/PFGE D/SCC*mec* V_T_/PVL-positive and the isolate from follow-up sampling after the first decolonization also had the same PFGE type.

In contrast, of the 233 patients participating in both surveys, 18 (64%) of 28 with MSSA colonization in the first survey still had MSSA colonization in the second survey. Of the 36 MSSA isolates from 18 subjects with paired isolates, 10 PFGE patterns were identified. Paired isolates from the same patient were indistinguishable for 17 of the 18 patients.

During the study period, two (1.24%) of 161 patients in CGMH, one with MRSA colonization (9.1%), developed MRSA infection. The clinical isolate was not preserved and not available for comparison of genetic relatedness with the colonization isolate. With decolonization procedures applied to those with MRSA colonization, no significant difference was found between the patients with and without MRSA colonization for subsequent MRSA infection.

## Discussion

Results from this study showed that the mean one-time point MRSA carriage rate of patients receiving hemodialysis in northern Taiwan was 3.8%, and the rate was up to 6.9% for those receiving two-time point surveys. For patients receiving hemodialysis, the nasal MRSA carriage rate was different, with a wide range, in different countries and regions, and also different with the different number of samplings. In previous reports from Taiwan, the average one-time point MRSA carriage rate among patients receiving hemodialysis was 2.4% (of 509 patients) in a report from southern Taiwan [22], and the rate was up to 9.5% of 306 patients with two serial surveys in another study from northern Taiwan [23]. Both rates were comparable to those in the present study.

For one-time point survey, the rate of nasal MRSA carriage (3.8%) among the patients receiving hemodialysis in the present study was also similar to that of adult patients visiting emergency room (3.8% of 502 patients) [20] and that of adults for health examination (3.8% of 3098 adults) reported from Taiwan [24]. Since most of the patients receiving hemodialysis came from community settings and both out-patient hemodialysis clinics were located in independent spaces of both hospitals, these patients were not frequently exposed to the patients with at-risk for MRSA acquisition, such as patients in intensive care units (ICU).

In the present study, we also found that no significant difference for nasal *S. aureus*, either MSSA or MRSA, carriage rate was found between patients treated at the medical center and the local hospital. Though the patients receiving hemodialysis may be associated with an increased risk of MSSA or MRSA colonization [20,25,26], no additional significant risk factor for *S. aureus* was identified among patients receiving hemodialysis in the present study. No significant difference was found, either, between the isolates from both hospitals in terms of antibiotics susceptibilities and molecular characteristics. These results suggest that the characteristics of the patients as well as the environments of out-patient hemodialysis at a local hospital and a medical center in northern Taiwan were similar.

Molecular characterization of all MRSA isolates in the present study showed that 70% of the isolates shared a common characteristics of endemic CA-MRSA clones (ST 59 or its single locus variant, 338) in Taiwan [4]. The remaining 6 isolates carried type IV or V SCC*mec*, also suggesting community strains [27]. The scenario of community strains being transmitted to healthcare facilities was indicated. The patient with the isolate of ST 30 had a travel history to other Asian countries, where the strains of ST 30 prevailed in the community; whether the patient acquired the isolate abroad needs further studies.

Scanty studies assessed the effect of MRSA decolonization for patients receiving hemodialysis and indicated that these patients might benefit from decolonization, though repeated courses of treatment are needed and the effects are modest [28]. In the present study, only one of 9 MRSA colonizers without successful elimination was persistent colonizer; in contrast, without elimination procedures, nearly two-thirds of 28 MSSA colonizations were persistent. Furthermore, with successful decolonization for most colonizers, no significant difference was found between the patients with and without MRSA colonization for subsequent MRSA infection, though the size of the patient’s number was small. These findings support that patients receiving hemodialysis with MRSA colonization may be successfully decolonized with intranasal mupirocin treatment plus chlorhexidine bath and might benefit from the reduction of subsequent MRSA infection to a rate comparable to that among those without MRSA colonization. The issue whether decolonization of MRSA may reduce subsequent infection in this population needs a large scale randomized control study.

There are several limitations for the present study. First, less than 60%, lower than expected, of the patients receiving hemodialysis in CGMH participated in this study, which reduced the size of case number for evaluation and indirectly affected the analysis of statistic significance. Second, from each study subject, samplings for MRSA detection were obtained only from one site (nares) once or twice, so some MRSA colonizing patients might be undetected [29]. Third, for each patient receiving de-colonization therapy, only one follow-up sampling was obtained one week after treatment, which might be inadequate for the proof of successful decolonization [30,31]. It is an issue that how many follow-up samplings and how long the observation duration are adequate for the proof of successful decolonization.

## Conclusions

A substantial proportion of patients receiving hemodialysis in Taiwan had MRSA colonization, mostly genetically community strains. No additional significant factor for MRSA carriage was identified among these patients. Decolonization procedures with intranasal mupirocin treatment plus chlorhexidine bath for 5 days may effectively eliminate MRSA colonization and might reduce subsequent MRSA infection in these patients.

## Abbreviations

MRSA: Methicillin-resistant *Staphylococcus aureus*; MSSA: Methicillin-sensitive *Staphylococcus aureus*; PFGE: Pulsed-field gel electrophoresis; SCC: Staphylococcal chromosomal cassette; MLST: Multilocus sequence type; PVL: Panton-Valentine leukocidin.

## Competing interests

The authors have no conflicts of interest relevant to this article to disclose.

## Authors’ contributions

YCK: laboratory performance, acquisition of data, analysis and interpretation of data, drafting the manuscript. WCT: laboratory performance, acquisition and analysis of data. CCY and JHK: conception and design, acquisition of data. YCH: conception and design, analysis and interpretation of data, modifying and revising the manuscript. All authors read and approved the final manuscript.

## Pre-publication history

The pre-publication history for this paper can be accessed here:

http://www.biomedcentral.com/1471-2334/12/284/prepub
